# Reply: Right ventricular remodeling in athletes and *crista supraventricularis* pattern

**DOI:** 10.1002/clc.23382

**Published:** 2020-05-22

**Authors:** Amelia Carro, María Sanz‐de la Garza, Stefano Caselli

**Affiliations:** ^1^ Corvilud Institute Asturias Spain; ^2^ Cardiovascular Institute, Hospital Clínic, IDIBAPS Barcelona Spain; ^3^ Cardiovascular Center Zürich Zürich Switzerland

To the Editor,

We are more than honored to receive the comments by Martinez‐Sellés et al^1^ regarding potential implications of the presence of a *crista supraventricularis* pattern in athletes. We do agree that different definitions of incomplete right bundle branch block (RBBB) have historically been applied in the field of athlete's electrocardiogram (ECG), hindering a homogeneous assessment of the benign or pathologic nature of its presence. In fact, many factors contribute to surface ECG expression, such as distance between the chest wall and the heart, race or body fat, among others.^2^ In fact, current international recommendations for ECG interpretation in athletes are not intended to establish diagnosis of cardiac disease; they serve as a screening tool that identifies high‐risk patterns that might be carriers or affected individuals of conditions associated with sudden cardiac death.^3^ However, exercise‐induced cardiac remodeling might develop ECG patterns that resemble the mentioned high‐risk features, and deserve additional assessment, as explained in our review.^4^

We thank the authors for introducing the concept of *crista supraventricularis* pattern in the sports cardiology field, where it had not been previously addressed. This pattern is characterized by a QRS width ≤ 100 ms with an RSR morphology in lead V1. *Crista supraventricularis* comprises the muscular structure separating anterosuperior tricuspid leaflet from pulmonary valve. It is shown that subendocardial *crista supraventricularis* Purkinje network is closely connected with the large septal network present along the entire septum except at a free zone below ventricular valves.^5^ This explains the ECG pattern described and the diseases that usually develop this trait. Most of them include septal defects or right ventricular hypertrophy. This ECG pattern has been shown to partially resolve after surgical correction of the defect^6^ suggesting that it is directly associated with a disproportional right ventricular overload. This reversibility after correction of the defect hypothesizes the transient nature of electrophysiological alterations; furthermore, it serves as a rationale for deepen into incomplete RBBB in athletes. Although not previously associated with exercise‐induced cardiac adaptations or even athlete's heart, the *crista supraventriculari*s pattern might play a role for some of the incomplete RBBB described in this population. An excess or unbalanced afterload is the stimulus for excessive right ventricular remodeling in highly trained athletes.^4^

Therefore, the prevalence of *crista supraventricularis* pattern in athletes might be subjected to further research, as it could be a marker of excessive right ventricular remodeling.
